# The global burden of metabolic and alcohol-associated liver disease (MetALD)

**DOI:** 10.1097/HC9.0000000000001053

**Published:** 2026-09-18

**Authors:** Gurmehr Brar, Brian P. Lee

**Affiliations:** 1 Department of Medicine, University of Southern California, Los Angeles, California, USA; 2 Division of Gastroenterology and Liver Diseases, University of Southern California, Los Angeles, California, USA

**Keywords:** alcohol, ALD, epidemiology, MASLD, SLD

## Abstract

Chronic liver disease is a leading cause of global morbidity and mortality, with an evolving etiologic landscape driven by rising metabolic dysfunction and persistent alcohol use. The introduction of metabolic and alcohol-associated liver disease (MetALD) recognizes the coexistence of these exposures as dual drivers of liver-related outcomes as a distinct clinical entity. This review synthesizes current evidence on the global epidemiology, clinical outcomes, and public health implications of MetALD. Current evidence suggests that ~10%–20% of individuals worldwide with hepatic steatosis meet criteria for MetALD. Emerging data demonstrate that the combination of cardiometabolic risk factors and alcohol is associated with more aggressive liver disease, including accelerated fibrosis progression, increased risk of cirrhosis and hepatic decompensation, and higher incidence of hepatocellular carcinoma compared with either exposure alone. In addition to major adverse liver outcomes, individuals with MetALD experience increased all-cause mortality, reflecting both progressive liver disease and the systemic effects of metabolic dysfunction and alcohol use. These factors contribute substantially to global disability-adjusted life years, particularly among working-age populations. The burden of MetALD varies across regions, reflecting differences in metabolic risk, alcohol consumption, and socioeconomic determinants, and is expected to rise further in both high- and middle-income settings. Despite its growing clinical relevance, the global burden of MetALD remains incompletely characterized due to limitations in standardized definitions, data availability, and ascertainment of alcohol use. Improved recognition, harmonized definitions, and integrated clinical and public health strategies are essential to better characterize and mitigate its impact on global liver health.

## INTRODUCTION

Chronic liver disease (CLD) is a leading cause of morbidity and mortality.^1^ Recent estimates indicate that cirrhosis and liver cancer together account for more than 2 million deaths annually and rank among the leading causes of years of life lost.^1^ Historically, CLD was driven by viral hepatitis and alcohol-associated liver disease (ALD), with regional variation in the contributions of these etiologies.^2^ However, the etiologic landscape has shifted over the past 2 decades as widespread vaccination and antiviral therapies have reduced the burden of viral hepatitis while rising rates of obesity, type 2 diabetes, and metabolic syndrome have driven a parallel increase in metabolic liver disease.^2^ Metabolic dysfunction–associated steatotic liver disease (MASLD) has emerged as the most common cause of CLD globally, affecting over one-third of adults and increasing by 50% over the past 2 decades.^3^ Concurrently, harmful alcohol consumption remains prevalent, resulting in a growing population exposed to both metabolic and alcohol-associated risk factors for CLD.^4^

Recognizing the limitations of previous nomenclature, a 2023 international consensus introduced terminology to describe the spectrum of steatotic liver disease (SLD).^5^ Within this framework, a new category, metabolic and alcohol-associated liver disease (MetALD), was established to describe individuals with SLD who have both cardiometabolic risk factors (CMRF) and alcohol consumption above the threshold for MASLD yet lower than ALD.^5^ While CMRFs drive liver-related outcomes, such as fibrosis progression and liver decompensation, among individuals with MASLD, alcohol is the main driver of liver-related outcomes among individuals with ALD, even though the majority of individuals with ALD have CMRFs.^6^ MetALD is meant to reflect a population among whom CMRFs and alcohol both contribute meaningfully to liver-related outcomes. Emerging evidence suggests that the combined presence of these exposures may accelerate fibrosis progression and increase the risk of cirrhosis and hepatocellular carcinoma (HCC) compared with either risk factor alone.^7^,^8^ Despite its clinical and public health significance, the global epidemiology of MetALD remains incompletely characterized.

This review describes the current global burden of MetALD by (i) characterizing the epidemiology of MetALD, (ii) describing its clinical course and management, and (iii) outlining implications for public health and healthcare policy.

## DEFINITIONS, NOMENCLATURE, AND CONCEPTUAL FRAMEWORK

In 2023, a multisociety Delphi consensus statement was issued, renaming non-alcoholic steatohepatitis (NASH) and non-alcoholic fatty liver disease (NAFLD) to metabolic dysfunction–associated steatohepatitis (MASH) and MASLD.^5^ This change was driven by the desire to eliminate stigmatizing terminology and incorporate language that better reflects disease biology.^5^ MASLD is defined by hepatic steatosis in the presence of one or more CMRFs such as obesity, hypertension, hypertriglyceridemia, hyperlipidemia, and insulin resistance.^5^ In contrast, ALD encompasses a spectrum of liver disease ranging from steatosis to cirrhosis driven by alcohol consumption of >350 g/week for women and >420 g/week for men. This framework also introduces MetALD, which recognizes the coexistence of metabolic dysfunction and alcohol consumption as the joint drivers of liver disease. MetALD is defined as patients who meet criteria for MASLD in conjunction with alcohol intake of 140–350 g/week for women and 210–420 g/week for men.

## GLOBAL BURDEN OF METABOLIC DYSFUNCTION AND ALCOHOL USE

### Global epidemiology of metabolic dysfunction

Understanding the global burden of MetALD requires examination of the parallel epidemiology of its 2 principal drivers: metabolic dysfunction and alcohol use. From 1990 to 2019, the prevalence of metabolic dysfunction has increased substantially, driven largely by the increase in obesity, which now affects ~900 million adults worldwide.^9^ Type 2 diabetes has followed a similar trajectory, affecting nearly 600 million individuals globally.^10^ By 2050, 1 in 8 individuals are projected to have diabetes, with more than 80% of those cases occurring in low- and middle-income countries.^10^,^11^ Between 1990 and 2022, parts of Northern Africa, the Middle East, and South America have seen an absolute 5%–20% increase in obesity and now have a greater proportion of obese citizens than underweight.^9^,^12^ Importantly, regional and ethnic differences in metabolic dysfunction exist. South Asians, for example, develop insulin resistance at body mass indices (BMI) 3–5 kg/m^2^ lower than Europeans, while East Asians have greater abdominal visceral fat than Caucasians (23.9%, 95% CI 20.8–27.0 vs. 18.5%, 95% CI, 15.9–21.1) when controlling for BMI, making these populations higher risk for metabolic complications.^13^,^14^,^15^ Socioeconomic gradients further shape the distribution of disease burden, with lower socioeconomic status populations experiencing higher rates of metabolic disease due to reduced access to healthcare, lower health literacy, and greater reliance on calorically dense, processed diets.^16^,^17^

### Global alcohol consumption patterns

From 2010 to 2019, alcohol consumption has remained prevalent worldwide with an average alcohol per capita consumption (APC) of 5.7 L among adults.^4^ The highest rates are reported in Europe and the Americas, where more than 75% of adults consume alcohol, while 6% in the Middle East and 20%–30% of adults drink alcohol across Asia and Africa.^4^ Consumption has risen notably in Southeast Asia and Southern Europe, with increases in APC of 0.5 and 0.4 L.^4^ These shifts likely reflect evolving religious and cultural norms alongside changing economic conditions. In fact, national wealth is a strong predictor of alcohol use: in 2019, high-income countries had an average APC of 9.8 L compared with 4 L in low-income countries.^4^

Globally, men drink more than women, 52.2% compared with 35.4%, though this gap narrows considerably in higher-income countries, 76.9% compared with 64.1%.^4^ Several low- and middle-income nations have seen a convergence in male and female drinking patterns as economic prosperity has grown, and among adolescents aged 15–19, the gender gap has diminished even further.^4^ In the Western Pacific Region, the prevalence of female drinkers grew by 23% from 2000 to 2019, outpacing the growth in male drinkers of 21.1%. Heavy episodic drinking, defined as consuming at least 60 g of alcohol on one occasion in the last month, is employed globally by 38% of drinkers, 27% female and 45% male.^4^ Binge drinking is prevalent among adolescents as well, with 15% of adolescents reporting drunkenness within the last month.^4^ Taken together, these trends underscore the continued and evolving public health significance of alcohol as a driver of liver disease.

## PREVALENCE AND INCIDENCE OF METALD

### Population-based estimates

Estimating the true prevalence and incidence of MetALD remains challenging, owing to the 2023 adoption of standardized nomenclature and considerable variability in diagnostic approaches across studies. Large cohort studies suggest that ~10%–20% of individuals with hepatic steatosis fall into this dual exposure category, though these figures are likely conservative given the underreporting of alcohol intake and the historical tendency to assign a single primary etiology.^6^,^18^,^19^,^20^ A study using the United States National Health and Nutrition Examination Survey (NHANES) data from 1988 to 2023 found that self-reported alcohol intake significantly underestimates the prevalence and mortality risks of alcohol-associated liver diseases and, by calibrating this data to national consumption levels, found that from 2021 to 2023 the adjusted prevalence of MetALD was 4.10%, decreasing to 2.14% when unadjusted.^21^

Available estimates of MetALD prevalence derive from methodologically diverse sources, each with distinct strengths and limitations. An imaging-based study using transient elastography from NHANES 2017–2020 found an age-adjusted MetALD prevalence of 2.56%, 13.27% of whom had significant fibrosis (liver stiffness >8.6 kPa), and 5.47% had advanced fibrosis (liver stiffness >13.1 kPa).^22^ Similarly, a cross-sectional analysis of over 40,000 patients from the United Kingdom Biobank used liver MRI to estimate a MetALD prevalence of 7.9%.^19^ Notably, these imaging-based studies are limited by reliance on self-reported alcohol intake, which is often underreported, as well as the absence of liver biopsy to more accurately assess the degree of fibrosis. A meta-analysis pooled 44 studies comprising 11,282,575 participants and estimated a global SLD prevalence of 37.5% (95% CI 31.4–44.1), with subtype prevalences of 33.6% (95% CI 28.1–39.5) for MASLD, 4.1% (95% CI 3.1–5.3) for MetALD, and 2.2% (95% CI 1.5–3.1) for ALD. Prevalence rose to 70.2% among individuals with diabetes and 70.7% among overweight or obese participants.^23^ Importantly, the constituent studies applied differing steatosis thresholds and imaging modalities, and classification of MetALD and ALD depended on self-reported alcohol intake and is therefore likely to be underestimated.^23^ A retrospective cohort study in the United Kingdom utilizing liver biopsy reclassified patients from 1999 to 2024 into the new SLD framework, finding that 12% of patients met criteria for MetALD.^24^ Likewise, a prospective study in Denmark from 2013 to 2018 used liver biopsy to determine that 24% of patients with SLD have MetALD.^20^ Given that liver biopsy is generally reserved for patients with an uncertain diagnosis or more advanced liver disease, biopsy-based studies may overestimate the severity of liver disease and therefore limit generalizability. Across methodologies, a consistent theme emerges: a substantial proportion of patients with SLD have overlapping metabolic and alcohol-associated exposures. Heterogeneity in case definitions, diagnostic tools, and ascertainment strategies nonetheless limits direct comparisons across studies and likely contributes to the wide range of prevalence estimates reported in the literature (Table 1).

**TABLE 1:** Prevalence estimates of MetALD across global cohorts

Study	Country	Design	Sample size	Diagnostic method	MetALD prevalence	Key limitations
NHANES 1988–2023 (Younossi et al)^21^	USA	Cross-sectional, national health survey of the general population	41,100	Self-reported alcohol calibrated to national consumption levels	4.10% (95% CI 3.38–4.82)	Self-reported alcohol use, no liver biopsy
NHANES 2017–2020 (Kalligeros et al)^22^	USA	Cross-sectional, national health survey of the general population	9698	Transient elastography	2.56% (95% CI 1.91–3.41)	Self-reported alcohol use, no liver biopsy
UK Biobank 2006–2010 (Schneider et al)^19^	UK	Cross-sectional, population biobank	40,534	Liver MRI	2.10% (no CI reported)	Self-reported alcohol use, no liver biopsy, and biobank volunteers may not represent the general population
UK retrospective cohort 1999–2023 (Pekarska et al)^24^	UK	Retrospective cohort of patients undergoing liver biopsy for SLD staging	784	Liver biopsy	12% (no CI reported)	Biopsy-selected population skews toward advanced disease; limited generalizability
Danish prospective cohort 2013–2018 (Israelson et al.)^20^	Denmark	Prospective cohort of patients with current or previous excessive alcohol use	446	Liver biopsy	24% (No CI reported)	Biopsy-selected population skews toward advanced disease; limited generalizability
National retrospective study 2015-2023 (Lin et al.)^34^	China	Multicenter retrospective cohort	501,017	Survey study and medical record review	3.49% (95% CI 3.38–3.60)	Retrospective design; diagnostic heterogeneity; no biopsy data
Retrospective Cohort 2004–2022 (Nakahata et al)^13^	Japan	Single-center retrospective cohort	184,463	Medical record review	2.9% (no CI reported)	Retrospective design; no biopsy data
Veterans Health Administration 2009–2021 (Ochoa-Allemant et al.)^48^	USA	Retrospective cohort of patients with hepatic steatosis	366,433	Medical record review	17.5% (no CI reported)	Male-predominant veteran population; limited generalizability to women and non-veterans; retrospective design

*Note:* Summary of population-based and clinical cohort studies reporting the prevalence of metabolic and alcohol-associated liver disease (MetALD) across geographic regions, illustrating the substantial heterogeneity in prevalence estimates attributable to differences in study design, diagnostic methodology, and case ascertainment strategies. Prevalence estimates are not directly comparable across studies, given differences in diagnostic thresholds, alcohol ascertainment methods, and study populations.

Abbreviations: CI, confidence interval; MASLD, metabolic dysfunction–associated steatotic liver disease; MetALD, metabolic and alcohol-associated liver disease; MRI, magnetic resonance imaging; NHANES, National Health and Nutrition Examination Survey; SLD, steatotic liver disease; UK, United Kingdom; USA, United States of America.

### Objective alcohol biomarkers and PEth-corrected estimates

Due to the unreliability of self-reported alcohol use, direct alcohol biomarkers are important in characterizing the epidemiology of MetALD. Phosphatidylethanol (PEth) is a phospholipid formed in the presence of ethanol, remains detectable for 2–4 weeks after consumption, and is more sensitive and specific than indirect markers of alcohol use.^25^,^26^ In a prospective cohort of overweight and obese adults and liver phenotyping by MRI, PEth discriminated MetALD from MASLD with an AUROC of 0.81 at a threshold of 25 ng/mL. Further, 16% of participants underreported their intake, and adding PEth to self-report increased MetALD and ALD diagnoses 4-fold and 3-fold, respectively.^26^ In a Danish prospective cohort of 2924 individuals at risk for SLD, 39.5% of participants in the alcohol-risk group and 11.1% in the metabolic-risk group underestimated intake relative to PEth, and 39.0% of those classified as having MASLD would be reclassified as MetALD or ALD depending on the PEth threshold applied.^27^ Taken together, PEth-corrected estimates suggest that the true prevalence of MetALD is severalfold higher than self-reporting indicates. Importantly, a PEth measurement reflects only recent intake and cannot capture cumulative or fluctuating exposure, and must be interpreted alongside a structured alcohol history.^25^,^28^

### Regional epidemiology

The burden of MetALD varies considerably across regions, shaped by differences in metabolic risk profiles and alcohol consumption patterns. In North America, high rates of obesity and alcohol use have created a substantial and growing population at risk for MetALD.^29^,^30^ Data from NHANES 2017–2023 estimates that among the 34% of American adults with SLD, 2% meet criteria for MetALD.^6^ National cohort data from Denmark and the United Kingdom report MetALD prevalence ranging from 8% to 24%, reflecting both Europe’s high rate of alcohol use as well as its well-characterized metabolic risk burden.^18^,^19^,^20^,^31^,^32^ Regional differences should not, however, be attributed solely to differences in alcohol exposure as the degree to which alcohol intake is underreported varies by region, reflecting differences in cultural and religious norms, survey instruments, and the extent to which objective biomarkers are used in routine care.^4^,^25^ Many European cohorts have incorporated PEth into clinical and research settings, which reclassifies a portion of MASLD as MetALD and increases prevalence relative to regions that rely on self-reported alcohol use alone.^26^,^27^ Conversely, where alcohol use carries greater social, religious, or legal sanction, self-reported intake is likely to be understated, and the low MetALD prevalence reported across parts of Asia, the Middle East, and North Africa may reflect this.^4^

In Asia, the epidemiology of SLD is shaped by metabolic and genetic susceptibilities, most notably the propensity to develop metabolic complications at a lower BMI.^33^ Although alcohol consumption has historically been lower across parts of the region, rising intake, especially among women, associated with rapid economic growth, suggests that the burden of MetALD may increase in the coming decades.^4^ In China, a national retrospective study estimated a MetALD prevalence of 3.5% in 2023, with higher rates observed among men and older adults.^34^ In Japan, a population-based study reported a MetALD prevalence of 2% with a comparable burden observed in both the general population and the lean (BMI<23 g/m^2^) subgroup.^13^ In Latin America and the Middle East, high prevalence of metabolic syndrome combined with variable alcohol use patterns creates region-specific risk profiles that remain incompletely characterized. Meanwhile, data from sub-Saharan Africa remains limited, representing a critical gap in the global epidemiology of MetALD.

### Temporal trends

**FIGURE 1:**
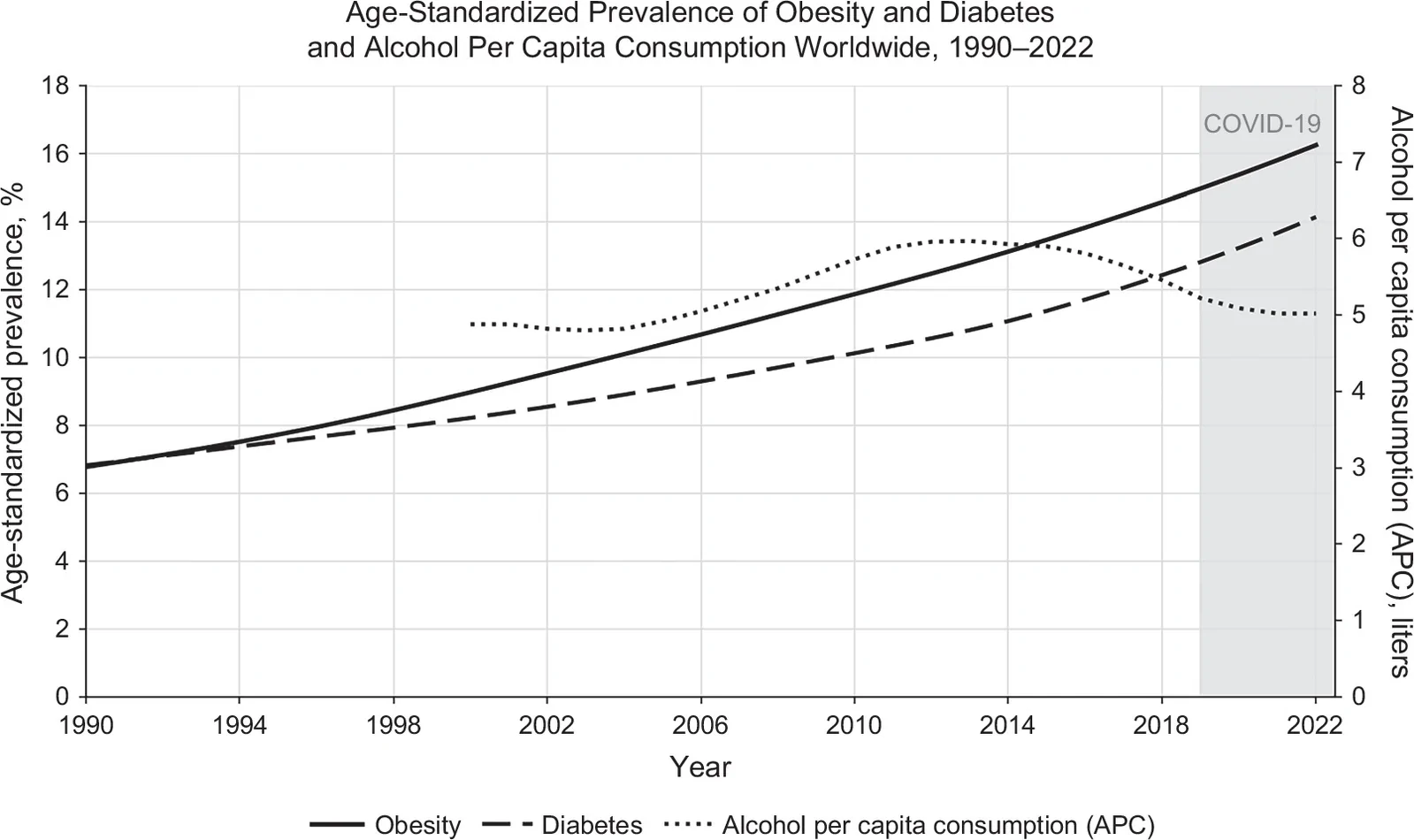
Age-standardized prevalence of obesity and diabetes and alcohol per capita consumption worldwide, 1990–2022. Global age-standardized prevalence of obesity (BMI ≥30 kg/m^2^) and diabetes, and alcohol per capita consumption (APC) from 1990 to 2022.^4^,^9^,^11^ Obesity and diabetes prevalence (left *x*-axis) are age-standardized estimates averaged across sexes among individuals 18 years and older. APC (right *x*-axis) reflects total recorded alcohol consumption in liters of pure alcohol per capita among individuals aged 15 years and older. The year is represented on the *y*-axis. The shaded region denotes the COVID-19 pandemic period (2019–2022). Abbreviations: APC, alcohol per capita; BMI, body mass index; COVID-19, coronavirus disease 2019.

Although longitudinal data specific to MetALD are limited, given the 2023 nomenclature shift, broader epidemiologic trends in alcohol consumption and metabolic dysfunction point toward a rising prevalence.^6^,^17^,^18^,^19^,^20^ Since 1999, there has been a rise in the global rates of obesity and diabetes, while alcohol consumption has remained relatively stable or increased in most regions (Figure 1).^4^,^9^,^10^ The COVID-19 pandemic further altered these patterns, accelerating risk factor accumulation across both domains. Several studies reported increased alcohol consumption, particularly among individuals with pre-existing high-risk drinking behaviors.^35^,^36^ In the United States, a survey study of over 1500 participants found a 14% increase in overall alcohol consumption and a 41% increase in heavy drinking for women from 2019 to 2020.^36^ In Japan, a national database study demonstrated a 22% relative increase in hospital admissions due to alcohol-associated liver and pancreatic disease from 2018 to 2020.^37^ Across Asia, while there was a decrease in overall alcohol consumption, there was an increase in alcohol use related to social isolation, psychological stress, and economic uncertainty.^38^ For instance, in India, there was a 57% relative increase in alcohol use among the poorest 30% of households in both rural and urban areas compared with a 28% increase for the richest 30% of households.^39^ Similarly, a United States survey study in 2020 also saw 36% of participants reporting increased alcohol use compared with the year prior, with household job loss increasing the odds of high-risk drinking by 41% and depressive symptoms increasing the odds by 5%.^38^ Concurrently, pandemic-era sedentary behavior and weight gain compounded metabolic risk across affected populations.^39^,^40^ A longitudinal analysis of over 200 Japanese participants from 2018 to 2020 saw an overall increase in sedentary time associated with increased visceral fat accumulation (β=3.85, 95% CI 1.22–6.49) with no change in diet or alcohol intake.^40^ Together, these trends suggest a growing MetALD burden and the need for early identification and prevention.

## DISEASE PROGRESSION AND CLINICAL OUTCOMES

**FIGURE 2:**
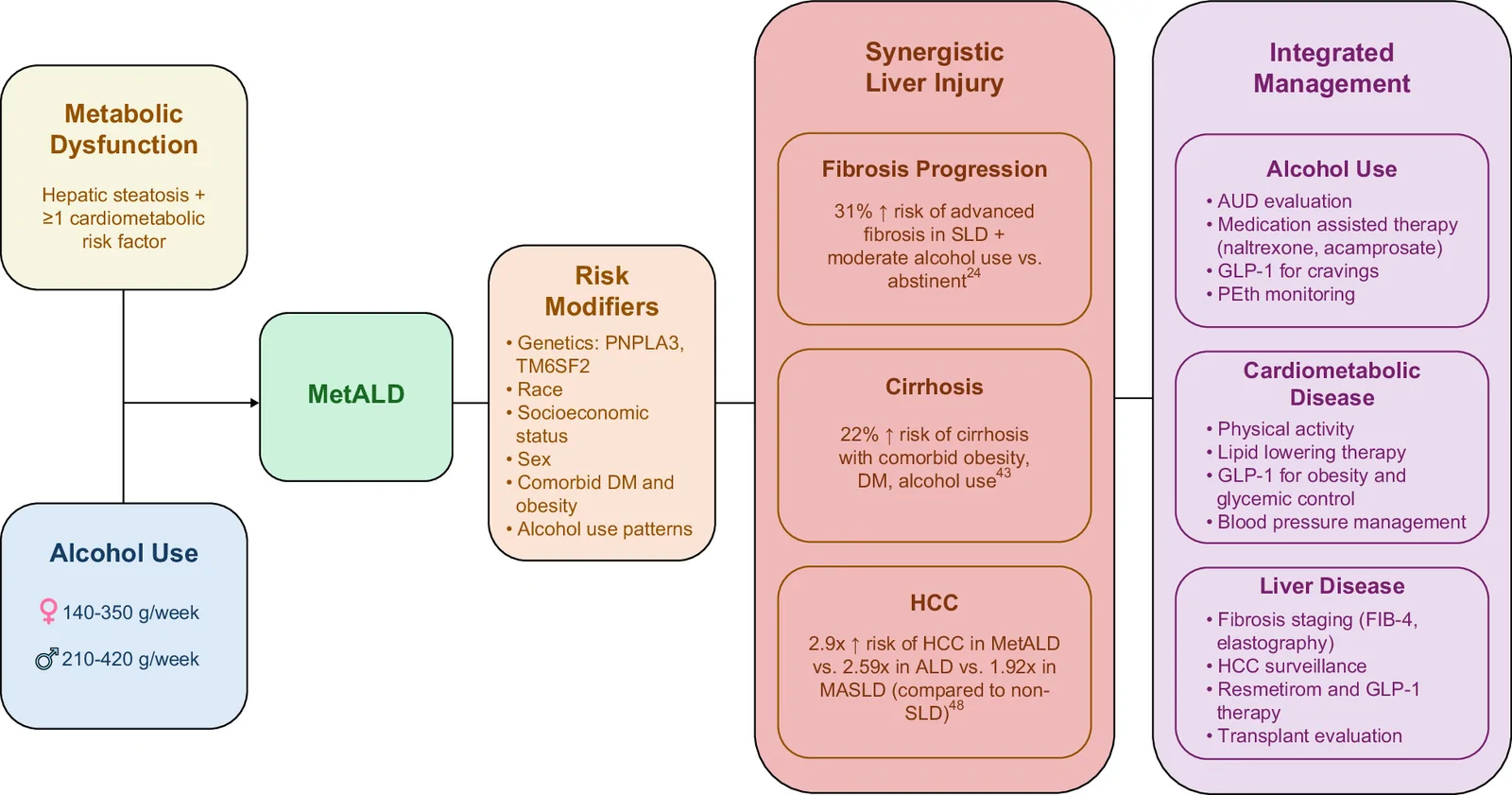
Conceptual framework of MetALD as a dual-driver disease process. Metabolic and alcohol-associated steatotic liver disease (MetALD) is defined as metabolic dysfunction (hepatic steatosis with ≥1 cardiometabolic risk factor) and alcohol intake of 140–350 g/week in women and 210–420 g/week in men. Metabolic dysfunction and alcohol use drive synergistic liver injury, leading to accelerated fibrosis progression, increased risk of cirrhosis, and amplified hepatocellular carcinoma (HCC) risk compared with either driver alone. Risk modifiers, including genetics (PNPLA3, TM6SF2), sex, race, socioeconomic status, and comorbid diabetes and obesity, further shape disease trajectory. Integrated multidisciplinary management addresses alcohol use disorder, cardiometabolic risk factors, and liver disease simultaneously. Abbreviations: AUD, alcohol use disorder; DM, diabetes mellitus; FIB-4, fibrosis-4 index; GLP-1, glucagon-like peptide-1; HCC, hepatocellular carcinoma; MASLD, metabolic dysfunction–associated steatotic liver disease; MetALD, metabolic dysfunction-associated steatotic liver disease with increased alcohol intake; PNPLA3, patatin-like phospholipase domain-containing protein 3; SLD, steatotic liver disease; TM6SF2, transmembrane 6 superfamily member 2.

The coexistence of metabolic dysfunction and alcohol consumption carries important implications for disease progression and clinical outcomes in MetALD. Emerging evidence suggests that individuals with dual metabolic and alcohol-associated risk factors may experience more aggressive liver disease compared with either exposure alone, supporting a model in which these insults interact to accelerate progression rather than act independently (Figure 2).^7^,^8^ These findings are summarized in Table 2.

**TABLE 2:** Comparative clinical outcomes across steatotic liver disease subtypes

Outcome	MASLD	MetALD	ALD
Primary driver of disease	Metabolic syndrome^6^	Synergy of metabolic syndrome and alcohol use^6^	Alcohol use^6^
Cirrhosis Incidence (cases per 100 person-years)	0.43 (95% CI 0.42–0.43)^47^	0.39 (95% CI 0.39–0.40) 47	0.66 (95% CI 0.65–0.66)^3^
Cirrhosis Incidence with obesity + DM (cases per 100 person-years)	*0.52 (95% CI 0.51–0.53); p<0.001*4^47^7	*0.49 (95% CI 0.48–0.51); p<0.001*47^47^	0.74 (95% CI 0.71–0.76); *p<0.0014^47^7*
HCC risk (vs non-SLD without CMRF)	HR 1.92 (95% CI 1.51–2.44)^52^	HR 2.91 (95% CI 2.11–4.03)^52^	HR 2.59 (95% CI 1.93–3.48)^52^
Liver-related mortality	Reference	HR 3.38 (95% CI 3.02–3.78)^48^	HR 6.99 (95% CI 6.08–8.04)^48^
Primary cause of death (no cirrhosis)	Cardiovascular disease [8.1% (95% CI 7.8–8.3)]^48^	Cardiovascular disease [7.5% (95% CI 7.2–7.9)]^48^	Cardiovascular disease [8.1% (95% CI 7.5–8.8)]^48^
Primary cause of death (cirrhosis)	Cardiovascular disease [17.3% (95% CI 15.6–20.1)] ^48^	Liver-related [17.7% (95% CI 15.6–20.1)]^48^	Liver-related [22.1% (95% CI, 18.4–26.3)]^48^

*Note:* Comparison of cirrhosis incidence, hepatocellular carcinoma (HCC) risk, liver-related mortality, and cause-specific mortality across metabolic dysfunction–associated steatotic liver disease (MASLD), metabolic and alcohol-associated liver disease (MetALD), and alcohol-associated liver disease (ALD), derived from population-based cohort studies. HCC risk and liver-related mortality hazard ratios are adjusted for relevant confounders in their respective source studies. The primary cause of death reflects the 10-year cumulative incidence among patients without and with cirrhosis separately.

Abbreviations: CI, confidence interval; CMRF, cardiometabolic risk factors; DM, type 2 diabetes mellitus; HR, hazard ratio; non-SLD, non-steatotic liver disease.

### Fibrosis progression

Fibrosis stage is a key determinant of long-term outcomes in CLD. A single-center study from the United Kingdom found that patients with advanced scarring face a significant escalation in risk compared with those with mild or no scarring (F0–F2 fibrosis).^24^ Specifically, individuals with F3 fibrosis exhibit a nearly 6-fold increase in liver-related mortality, while those with F4 fibrosis face an 11-fold increase in the risk of death, compared with those with F0–F2 fibrosis.^24^ This prognostic gap is similar when measuring major adverse liver outcomes (MALO) such as liver failure or cancer; the likelihood of experiencing such a life-threatening event is 4.56 times higher with F3 fibrosis and climbs to 9.54 times higher with F4 fibrosis, compared with those with F0–F2 fibrosis.^24^ Consequently, while a patient with early-stage disease may have a 5-year MALO risk as low as 1.8%, that risk surges to 25% once cirrhosis is present, underscoring that the physical stage of the liver is the ultimate determinant of a patient’s clinical trajectory.^24^

Dual exposure to metabolic dysfunction and alcohol appears to accelerate fibrosis as both risk factors independently promote hepatic steatosis, inflammation, and fibrogenesis through overlapping mechanisms, including oxidative stress, lipotoxicity, and activation of hepatic stellate cells.^7^,^8^ When present together, these pathways exert additive and potentially synergistic effects. A national cohort study in Finland following over 8000 participants found that in patients with pre-existing SLD, alcohol consumption presents a dose-dependent risk for both advanced liver disease and cancer.^41^ Consuming just 10–19 g/day of alcohol doubles the risk of progressing to advanced liver disease compared with lifetime abstainers with similar metabolic profiles.^41^ Further, among individuals with ALD, the presence of metabolic risk factors such as obesity and diabetes has been associated with more severe fibrosis and worse histologic outcomes.^41^,^42^,^43^,^44^,^45^ In one study, patients with ALD exceeding 110% of their ideal body weight exhibited significantly more liver necrosis and fibrosis.^45^ Subsequent research has demonstrated that concurrent diabetes and obesity in patients with ALD confer a 3-fold and 16-fold increase in liver-related mortality, respectively.^46^ While there is limited biopsy and population-level data on fibrosis stage and disease severity in patients with MetALD, these findings suggest that the interaction of alcohol and CMRF compounds the risk of either risk factor alone.

### Cirrhosis and hepatic decompensation

The consequences of metabolic and alcohol-associated injury extend beyond fibrosis to the development of cirrhosis and its complications. Patients with multiple CMRFs, namely obesity and diabetes, and alcohol use have a 22% higher risk of progressing to cirrhosis than those without comorbid obesity and diabetes.^47^ Existing data also suggest that the synergy of metabolic dysfunction and alcohol use may lead to a higher likelihood of developing cirrhosis than in metabolic dysfunction alone.^48^ Data from the Veterans Health Administration from 2009 to 2021 showed that cirrhosis was more prevalent among the MetALD group (5%) than the MASLD group (2.2%).^48^ Similarly, a longitudinal study from South Korea following 58,000 patients for 5 years found that patients with MASLD and alcohol use had a 31% higher relative chance of progressing from low to intermediate/high risk of advanced fibrosis on noninvasive liver stiffness measurements than those who were abstinent.^29^

Patients with MetALD are also more likely to experience hepatic decompensation. A meta-analysis of 24 cohort studies comprising 11,575,558 adults with SLD, of whom 9,801,312 had MASLD, and 1,774,246 had MetALD, found that MetALD carried a significantly higher risk than MASLD (HR 1.62, *p*=0.0086) of liver-related events.^49^ Once decompensated, patients with MetALD are also less likely to achieve recompensation than other etiologies. A retrospective cohort study at a single transplant center showed a liver recompensation rate of 3% in MetALD compared with 18% in ALD, emphasizing the severity of liver disease created by the synergy of alcohol use and metabolic dysfunction.^50^

### Hepatocellular carcinoma

The risk of HCC may also be further amplified in MetALD. Both alcohol and CMRFs are well-established independent risk factors for HCC, and their coexistence may further increase carcinogenic risk through chronic inflammation, oxidative DNA damage, and alterations in the hepatic microenvironment that promote tumor development.^51^,^52^,^53^ A meta-analysis including over 11 million individuals showed that patients with MetALD have a 33% greater risk of developing HCC compared with MASLD.^49^ Similarly, a prospective study of over 400,000 participants in Taiwan found that compared with people without SLD, patients with MetALD had a 2.9-fold greater risk of developing HCC, whereas patients with ALD and MASLD had a 2.59-fold and 1.92-fold greater risk.^47^ Notably, HCC may develop in this population even in the absence of advanced cirrhosis, particularly among individuals with long-standing metabolic disease.^53^ In a retrospective study of over 600,000 patients from the Veterans Analysis of Liver Disease cohort, the risk of HCC per 100,000 person-years in patients without cirrhosis with MetALD was 80.24 (95% CI 73.23–87.94), which was higher than MASLD (71.64, 95% CI 68.14–75.32) and lower than ALD (104.83, 95% CI 91.59–119.98).^50^ In non-cirrhotic MASLD patients, the majority of HCC is found incidentally, and, given that alcohol use is known to increase risk of HCC, current surveillance strategies may not fully capture patients at elevated risk with MetALD, warranting reassessment of screening thresholds.^52^

### Liver transplantation

MetALD is the third leading indication for liver transplantation in the United States.^55^ Analysis of UNOS data from 2002 to 2023 reveals that patients with MetALD experience a 2.9-fold higher rate of waitlist removal, a 10% higher relative risk of waitlist mortality, and 12% higher relative risk of graft failure when compared with ALD.^54^ This likely reflects a higher acuity population with greater risk of decompensation or death. At the same time, patients with MetALD demonstrate superior graft (12% higher relative risk for failure vs. 21% in MASLD) and overall survival (13% higher relative risk for all-cause mortality vs. 22% in MASLD) compared with MASLD.^54^ Post-transplant, obesity, diabetes, and alcohol relapse are associated with decreased long-term survival, meaning patients with MetALD face compounded risk across both the pre-transplant and post-transplant periods.^54^,^55^

### Mortality

Liver-related mortality has increased from 1990 to 2021, with global cirrhosis deaths increasing by nearly 40%.^1^ Data from NHANES 1988–2023 demonstrates a mortality rate of 8.74 per 1000 person-years in MetALD compared with 14.91 and 7.86 per 1000 person-years in ALD and MASLD.^21^ This increased mortality likely reflects both accelerated liver disease and the broader systemic consequences of metabolic syndrome and alcohol use, including cardiovascular disease and malignancy.^56^ In fact, a retrospective cohort study of 360,000 U.S. veterans reveals that in those with non-cirrhotic MetALD, the risk of liver-related death is 3.38 times higher than in MASLD (0.19 vs. 0.04 per 100 person-years).^48^ Once cirrhosis is present, liver-related mortality becomes a dominant threat for MetALD (17.7%) and ALD (22.1%) patients, whereas MASLD patients remain significantly more likely to die from cardiovascular events (17.3%) than liver complications (9.2%).^48^

## SPECIAL POPULATIONS AND MODIFIERS OF RISK

### Sex-based differences

Sex-based differences influence both susceptibility to and progression of MetALD. Women appear more vulnerable to alcohol-associated liver injury at lower levels of consumption than men, a phenomenon attributable to differences in alcohol metabolism, body composition, and hormonal influences.^57^,^58^ Similarly, metabolic risk profiles also differ by sex. Women are more likely to develop central adiposity and insulin resistance following menopause, whereas men tend to have higher baseline rates of alcohol consumption and earlier onset of metabolic dysfunction.^59^,^60^ Data from NHANES III show that while the prevalence of all SLD phenotypes is higher in men, the risk of all-cause mortality is higher in women.^61^ Specifically, women with MetALD face an 83% higher hazard of mortality, suggesting that women experience more rapid progression of alcohol-associated liver disease and face a higher relative risk of liver-related complications at comparable levels of exposure, a disparity that may be compounded in the setting of concurrent metabolic dysfunction.^61^ Yet despite this heightened vulnerability, women remain underrepresented in liver disease cohorts, limiting the generalizability of existing data and emphasizing the need for sex-stratified analyses in future MetALD research.

### Genetic and ethnic modifiers

Genetic predisposition plays a critical role in modulating susceptibility to liver injury in the setting of metabolic dysfunction and alcohol use. Among the most well-characterized genetic variants is patatin-like phospholipase domain-containing protein 3 (PNPLA3), which has been associated with increased hepatic fat accumulation, inflammation, fibrosis, and risk of HCC.^62^ In 2008, a genome-wide association study on hepatic steatosis found that individuals of Hispanic ancestry carry the risk allele at higher rates and demonstrate increased susceptibility to SLD and fibrosis, whereas individuals of African ancestry have lower risk allele frequencies and relatively lower rates of steatosis despite comparable metabolic profiles.^62^ Data from subsequent genome sequencing projects confirmed these patterns on a global scale, finding that the risk allele frequency is highest in Central and South America (~50%), followed by East Asia (35%–45%), South Asia (24%–30%), Europe (23%), and Sub-Saharan Africa (12%).^63^,^64^ Importantly, the impact of this variant appears to be amplified in the presence of both CMRFs and alcohol consumption, suggesting a potential interaction between genetic susceptibility and environmental exposures.^65^,^66^

Similarly, an exome-wide association study identified a transmembrane 6 superfamily member 2 (*TM6SF2*) polymorphism that is associated with increased hepatic triglyceride content and increased risk of progressive liver disease.^67^ The presence of the variant that confers increased susceptibility to SLD was higher in European ancestry (7.2%) than in African American (3.4%) or Hispanic (4.7%).^69^,^70^ Subsequent studies including Asian populations have found that the variant is also prevalent in East Asian individuals, up to 34%, broadening the global relevance of this variant. Of note, the TM6SF2 risk allele has been found at higher frequencies among patients with lean metabolic liver disease compared with non-lean counterparts, a finding that may partly explain the well-described tendency of Asian populations to develop metabolic liver disease at lower BMI.^68^,^69^

### Younger adults and emerging trends

Young adults represent an increasingly important and underrecognized population in the epidemiology of MetALD. Rising rates of obesity and diabetes in adolescents and young adults, combined with evolving patterns of alcohol use, including increased binge drinking, have created conditions favorable to the earlier onset of liver disease.^4^,^12^ In 2022, ~159 million adolescents were living with obesity, with a projected increase to 390 million by 2050, and 14.6 million were living with diabetes.^70^,^71^ Contemporaneously, binge drinking has increased among adolescents, with 15% reporting drunkenness within the last month.^4^ As a result, ALD and liver-related mortality are increasing among younger individuals, raising concerns about a shifting age distribution of disease burden.^72^ A comprehensive analysis of over 430,000 U.S. death certificates showed that between 2018 and 2022, there was an annual 19.5% increase in cirrhosis mortality among those aged 25–44 with ALD.^72^ This early onset of metabolic dysfunction and alcohol exposure may lead to a longer duration of cumulative liver injury, increasing the lifetime risk of advanced fibrosis, cirrhosis, and HCC. In addition, younger individuals with dual-risk factors may experience substantial long-term health and socioeconomic consequences, including reduced productivity and increased health care utilization, estimated to cost a total of $2.6 trillion international dollars between 2021 and 2050.^73^ These trends highlight the need for early identification and targeted prevention strategies in younger populations at risk for MetALD.

## CLINICAL AND PUBLIC HEALTH IMPLICATIONS

The recognition of MetALD has important implications for public health, clinical practice, and health policy. Because metabolic dysfunction and harmful alcohol use share overlapping drivers and frequently co-occur, integrated clinical approaches to risk reduction are essential. Lifestyle interventions targeting obesity, insulin resistance, and physical inactivity should be coupled with evidence-based strategies to reduce harmful alcohol consumption, such as medication-assisted treatment (MAT), including acamprosate and naltrexone.^74^ Recent pharmacologic developments offer additional promise for the MetALD population. Resmetirom, a thyroid hormone receptor beta agonist approved for the treatment of MASLD, demonstrated comparable reductions of ≥30% in liver fibrosis among patients in the MetALD subset and the broader MASLD cohort in the MAESTRO-NASH clinical trial, suggesting potential applicability across the SLD spectrum, although this was among a small subset of participants that would need confirmation in future trials.^75^ Glucagon-like peptide-1 (GLP-1) receptor agonists, such as semaglutide, have shown efficacy in reducing liver fibrosis in 36.8% of patients with biopsy-proven MASH and F2–3 fibrosis, while separately showing reductions in alcohol consumption and cravings; specifically, treatment with semaglutide (vs. placebo) significantly reduced drinks per drinking day (β=−0.41, 95% CI −0.73 to −0.09) and weekly alcohol craving (β=−0.39; 95% CI −0.73 to −0.06) among individuals with alcohol use disorder in the general population.^76^,^77^ This effect has since been substantiated at therapeutic doses. In a 26-week randomized, double-blind, placebo-controlled trial of 108 treatment-seeking adults with moderate-to-severe alcohol use disorder and obesity, all of whom received cognitive behavioral therapy, once-weekly subcutaneous semaglutide titrated to 2.4 mg reduced heavy drinking days by 41.1 percentage points versus 26.4 percentage points with placebo (95% CI −22.0 to −5.4), with concordant reductions in total alcohol consumption and drinks per drinking day and with greater improvements in body weight, waist circumference, and glycated hemoglobin.^78^ This positions GLP-1 receptor agonists as particularly compelling therapeutics for patients with MetALD, although prospective trials in this population are needed.

Fibroblast growth factor 21 (FGF21) analogs represent a second class with plausible dual activity in MetALD. FGF21 is a hepatokine that improves insulin sensitivity and reduces hepatic lipotoxicity, and in rodent and non-human primate models, it also suppresses alcohol preference through an amygdalo-striatal circuit, consistent with a liver–brain feedback loop regulating nutrient- and alcohol-seeking behavior.^79^ In phase 2b trials in MASH with F2–F3 fibrosis, the FGF21 analogs pegozafermin and efruxifermin improved fibrosis by at least one stage without worsening steatohepatitis, and pooled analyses of randomized trials of FGF21 analogs support a consistent antifibrotic effect. Human genetic data extend this rationale to the alcohol axis: in a Mendelian randomization analysis, genetically higher FGF21 signaling was associated with less problematic alcohol use and lower risk of alcohol-associated liver disease (OR 0.54, 95% CI 0.42–0.70), with effects mediated through reduced drinking rather than through hepatic fat alone, and with no comparable signal for other MASLD targets such as PNPLA3 or HSD17B13.^80^ FGF21 analogs merit dedicated evaluation in MetALD, where an agent that simultaneously attenuates alcohol intake and hepatic fibrogenesis would be of particular value.

Screening and early detection strategies must also evolve to reflect the dual-risk nature of MetALD. Current approaches assess metabolic risk and alcohol use independently, which may lead to under-recognition of patients with combined exposures. Systematically integrating alcohol use assessment into MASLD evaluation and metabolic risk screening into ALD evaluation would improve the identification of high-risk individuals. Noninvasive fibrosis assessment tools, including serum biomarkers and elastography, provide practical opportunities for earlier detection of advanced disease and should be applied with a lower threshold in those with dual-risk factors.^81^ A prospective community-based cohort of overweight and obese adults utilized a stepwise pathway to identify suspected MetALD patients using cardiometabolic criteria and self-reported alcohol intake in the MetALD range or a PEth level ≥25 ng/mL, then applied FIB-4 followed by elastography, diagnosing MetALD in 15.7% of those screened.^82^ An international expert panel has correspondingly proposed a MetALD-specific pathway that couples structured alcohol assessment, using validated instruments such as AUDIT-C supplemented by direct biomarkers, with conventional noninvasive fibrosis stratification, and that routes patients concurrently rather than sequentially to hepatology, addiction, and cardiometabolic care.^84^ Integrated and collaborative care models that co-locate treatment of alcohol use disorder with liver and metabolic care are the natural delivery vehicle for these pathways and have been associated with improved engagement and outcomes across the spectrum of alcohol-associated liver disease.^74^ Prospective evaluation of whether such pathways change outcomes in MetALD is needed.

At the population level, coordinated prevention efforts addressing diet, physical activity, alcohol availability, and alcohol marketing represent the most scalable opportunity to reduce upstream disease burden. From a health systems perspective, the economic impact of MetALD is likely substantial, encompassing direct costs such as hospitalizations, outpatient care, and liver transplantation, as well as indirect costs from lost productivity and premature mortality.^16^

## KNOWLEDGE GAPS AND FUTURE DIRECTIONS

Despite growing recognition of MetALD, knowledge gaps and methodological challenges remain. First, most existing epidemiologic datasets predate the introduction of standardized MetALD nomenclature and lack the ability to reliably identify individuals with MetALD. The development of standardized registries that incorporate the updated framework will enable more accurate surveillance and facilitate comparisons across populations. Second, reliance on self-reported alcohol consumption introduces systematic measurement error through recall bias and underreporting driven by stigma and cultural norms, likely resulting in both misclassification of patients and underestimation of alcohol’s contribution to liver disease burden. Third, alcohol intake is not stable within individuals. Drinking fluctuates with life circumstances, psychological distress, treatment, intercurrent illness, and season. Prospective data indicate substantial migration between SLD categories over short intervals: ~36% of individuals initially classified as MetALD were reclassified as MASLD or ALD within 6 months, about 32% of those with ALD moved toward MetALD or MASLD, and roughly 11% of those with MASLD were reclassified.^83^ Cumulative lifetime exposure is necessary for accurate estimation of both prevalence and risk, and SLD may be better conceptualized as a dynamic continuum along which individuals move than as a set of fixed categories.^25^,^83^ Fourth, there are geographic disparities in available data as the majority of epidemiologic liver disease research has been conducted in high-income countries, leaving gaps in regions where the burden of metabolic disease is rising most rapidly, particularly across sub-Saharan Africa, South Asia, and parts of Latin America.

Advances in biomarkers and noninvasive diagnostic tools also hold promise for improving the identification and risk stratification of patients with MetALD. The integration of serum biomarkers, imaging modalities, and clinical risk scores may allow for earlier detection of advanced fibrosis and more precise monitoring of disease progression. Improved risk stratification will be essential for guiding targeted interventions and optimizing resource allocation. In addition, global surveillance initiatives that incorporate both metabolic and alcohol-associated risk factors are needed to better capture the evolving burden of liver disease. Collaborative, multinational research efforts will be critical for generating high-quality data and informing evidence-based policy and clinical decision-making.

## CONCLUSION

MetALD represents an important and underrecognized phenotype of SLD arising from the convergence of 2 major global health challenges: metabolic dysfunction and harmful alcohol use. This review highlights the growing global burden of MetALD, including its epidemiology, clinical outcomes, and contribution to liver-related mortality. Emerging evidence suggests that the coexistence of metabolic and alcohol-associated risk factors is associated with more aggressive disease and worse outcomes than either exposure alone. As the prevalence of obesity, diabetes, and alcohol consumption continues to rise, the population at risk for MetALD is likely to expand, although current estimates likely underestimate the true impact of MetALD due to limitations in classification, data availability, and study design. Addressing this burden will require integrated clinical and public health strategies, improved surveillance, and coordinated efforts among clinicians, researchers, and policymakers.
